# A Randomized Trial of Liposomal Prednisolone (LIPMAT) to Enhance Radiocephalic Fistula Maturation: A Pilot Study

**DOI:** 10.1016/j.ekir.2020.05.030

**Published:** 2020-06-05

**Authors:** Bram M. Voorzaat, K.E.A. van der Bogt, Taisiya Bezhaeva, Jan van Schaik, Daniel Eefting, Karien van der Putten, Roos C. van Nieuwenhuizen, Johannes O. Groeneveld, Ellen K. Hoogeveen, Irene M. van der Meer, Randolph G. Statius van Eps, Liffert Vogt, Laurens Huisman, Bas A.Th.F. Gabreëls, Henk Boom, Cornelis A. Verburgh, Diederik Boon, Josbert M. Metselaar, Marcel C. Weijmer, Joris I. Rotmans

**Affiliations:** 1 Department of Internal Medicine, Leiden University Medical Center, Leiden, The Netherlands; 2 Department of Surgery, Leiden University Medical Center, Leiden, The Netherlands; 3 Department of Vascular Surgery, Haaglanden Medical Center, The Hague, The Netherlands; 4 Department of Nephrology, Tergooi Hospital, Hilversum, The Netherlands; 5 Department of Vascular Surgery, OLVG, Amsterdam, The Netherlands; 6 Department of Nephrology, OLVG, Amsterdam, The Netherlands; 7 Department of Nephrology, Jeroen Bosch Ziekenhuis, Hertogenbosch, The Netherlands; 8 Department of Nephrology, Haga Hospital, The Hague, The Netherlands; 9 Department of Vascular Surgery, Haga Hospital, The Hague, The Netherlands; 10 Department of Nephrology, Amsterdam Cardiovascular Sciences, Amsterdam University Medical Center, University of Amsterdam, Amsterdam, The Netherlands; 11 Department of Vascular Surgery, Amsterdam Cardiovascular Sciences, Amsterdam University Medical Center, University of Amsterdam, Amsterdam, The Netherlands; 12 Department of Nephrology, Alrijne Hospital, Leiderdorp, The Netherlands; 13 Department of Nephrology, Reinier de Graaf Hospital, Delft, The Netherlands; 14 Department of Nephrology, Spaarne Hospital, Haarlem, The Netherlands; 15 Department of Nephrology, Dijklander Hospital, Hoorn, The Netherlands; 16 Management Team, Enceladus Pharmaceuticals, Naarden, The Netherlands; 17 Institute for Experimental Molecular Imaging, RWTH Aachen University Clinic, Aachen, Germany

Patients on maintenance hemodialysis (HD) require a reliable vascular access; however, only half of newly created radiocephalic arteriovenous fistulas (RCAVF) can be used for HD without additional procedures to promote maturation and up 25% fail to provide adequate vascular access for HD.^1^ The need for subsequent creation of upper arm arteriovenous fistulas (AVFs) and arteriovenous grafts may decrease if maturation can be improved. Currently, no pharmacological treatments have been proven to improve clinical maturation of AVFs.

Although the underlying pathophysiology of nonmaturation is incompletely understood, impaired outward remodeling and neointimal hyperplasia in the venous outflow tract seem to contribute.^2^ Studies in murine and porcine models of AVF failure revealed a pronounced inflammatory response in the venous outflow tract in the early phase after AVF surgery.^3^ Recent studies suggest that this inflammatory response impairs AVF maturation.^4^

Pegylated liposomes have emerged as an attractive tool to facilitate selective delivery of drugs to inflamed tissues with a highly permeable microvasculature, where liposomes are being phagocytized by macrophages. It has a potent and long-lasting anti-inflammatory effect at sites of inflammation, while minimizing exposure of noninflamed tissues. In a murine model of AVF failure, we have demonstrated that liposomal prednisolone inhibits inflammation of the juxta-anastomotic vein and improves outward remodeling of the venous outflow tract.^5^

We hypothesized that maturation of RCAVFs in humans can be improved by inhibition of juxta-anastomotic inflammation using liposomal prednisolone. In the Liposomal Prednisolone to Improve Hemodialysis Fistula Maturation (LIPMAT) study, we aimed to assess if liposomal prednisolone improves maturation of RCAVFs and if it can be safely administered to patients with end-stage renal disease. The design of this multicenter randomized placebo-controlled trial has been reported earlier in detail,^6^ and methods are available in the Supplementary Materials.

## Results

### Study Population

**Figure 1:**
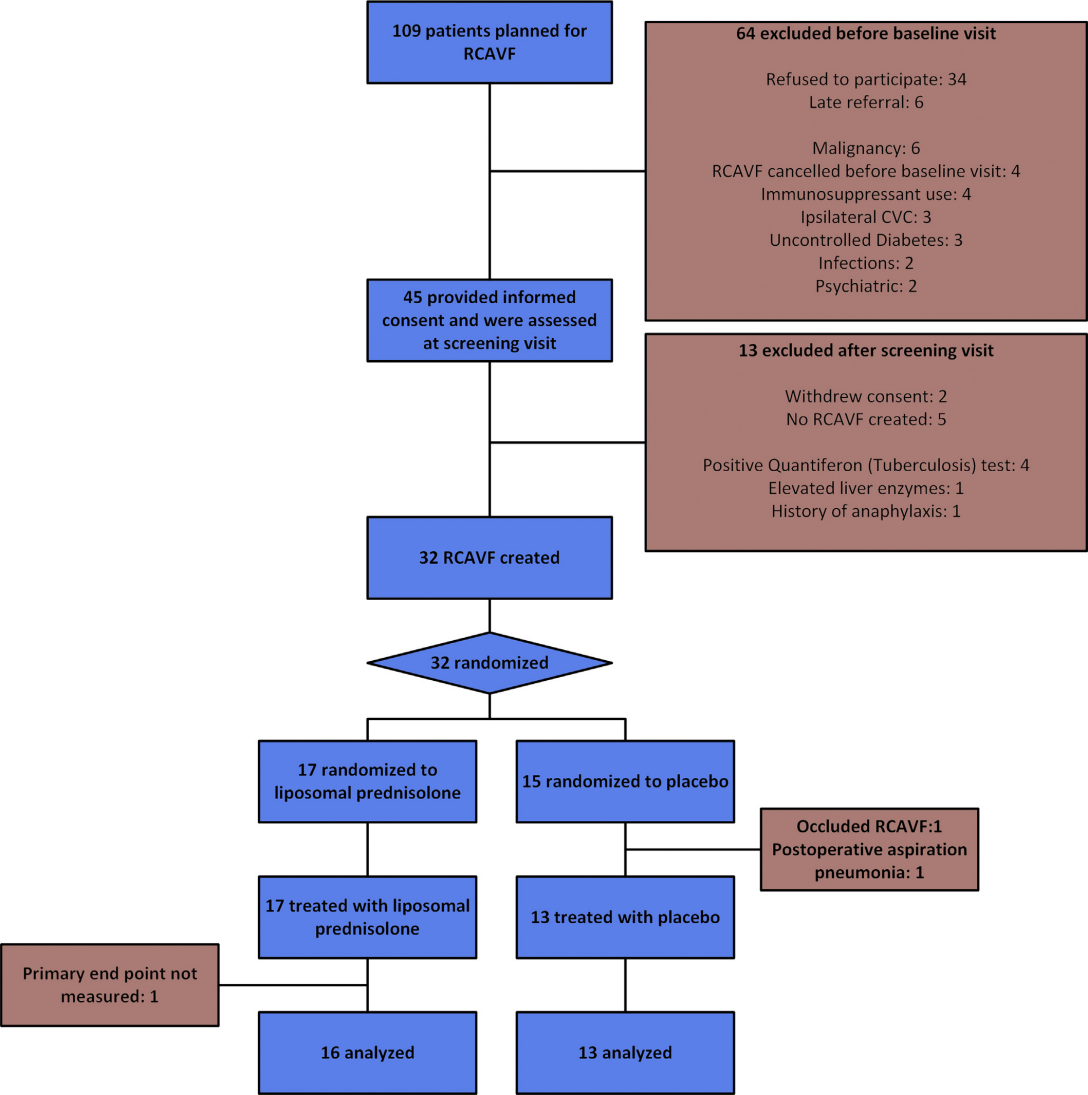
Study flowchart. CVC, central venous catheterization; RCAVF, radiocephalic arteriovenous fistula.

From April 2016 through May 2018, 109 patients were planned for RCAVF creation and assessed for study eligibility. A total of 64 patients were excluded for known exclusion criteria from their medical history (n = 24), not consenting to study participation (n = 34), or late referral (n = 6, Figure 1). Of the remaining 45 patients who provided informed consent, 32 were randomized (Table 1). Reasons for dropout are shown in Figure 1. After randomization, but before treatment, 2 patients experienced clinical events constituting exclusion criteria. The remaining 30 patients received the study treatment. The trial was stopped prematurely in May 2018 because of slow enrollment.

**Table 1:** Baseline characteristics of 29 patients in the LIPMAT study by treatment group

Baseline characteristics	Placebo (n = 13)	Liposomal prednisolone (n = 16)	Total (n = 29)
Age, yr	70 ± 8.5	65 ± 12	67 ± 11
Gender			
Female	5 (38)	1 (6)	6 (21)
Male	8 (62)	15 (94)	23 (79)
Race			
Caucasian	11 (85)	13 (81)	24 (83)
Hindustani Surinamese	1 (8)	2 (13)	3 (10)
Moroccan	0 (0)	1 (6)	1 (3)
Asian	1 (8)	0 (0)	1 (3)
Cause of renal failure			
Diabetes mellitus	4 (31)	6 (38)	10 (35)
Renal vascular disease	5 (39)	4 (25)	9 (31)
Glomerulonephritis	3 (23)	2 (13)	5 (17)
Interstitial nephropathy	1 (8)	2 (13)	3 (10)
Cystic kidney disease	0 (0)	2 (13)	2 (7)
Comorbidities			
Diabetes mellitus	7 (54)	7 (44)	14 (48)
Coronary artery disease	6 (46)	4 (25)	10 (35)
Peripheral artery disease	4 (31)	3 (19)	7 (24)
Cerebrovascular disease	5 (39)	4 (25)	9 (31)
Medication			
ACE inhibitor	1 (8)	6 (38)	7 (24)
Angiotensin 2 receptor blocker	8 (62)	5 (31)	13 (45)
Loop diuretic	8 (62)	9 (56)	17 (59)
Aldosterone receptor antagonist	0 (0)	1 (6)	1 (3)
Beta blocker	10 (77)	8 (50)	18 (62)
Calcium channel blocker	8 (62)	11 (69)	19 (66)
Platelet inhibitor	4 (31)	10 (63)	14 (48)
Anticoagulant	2 (15)	3 (19)	5 (17)
Vitamin D	12 (92)	13 (81)	25 (86)

ACE, angiotensin-converting enzyme; LIPMAT, Liposomal Prednisolone to Improve Hemodialysis Fistula Maturation.

Data are reported as mean ± SD or n (%).

### End Points

The primary end point was assessed in 29 patients. The distal cephalic diameter was 3.9 mm (95% confidence interval, 2.7–5.8 mm) in the placebo group and 3.7 mm (95% confidence interval, 3.0–5.3 mm) in the treatment group (*P* = 0.88). No significant results were observed for secondary end points (Table 2).

**Table 2:** Effect of liposomal prednisolone on primary and secondary end points in 29 patients in the LIPMAT study

End points	Placebo median (IQR)	Liposomal prednisolone median (IQR)	*P* (Mann-Whitney *U*)
6 wk			
Cephalic vein			
Juxta-anastomotic diameter, mm	3.9 (2.7–5.8)	3.7 (3.0–5.3)	0.88
Elbow diameter, mm	5.5 (4.7–6.7)	5.0 (4.0–6.1)	0.47
Mid upper arm diameter, mm	4.0 (2.3–5.3)	4.8 (4.1–5.4)	0.22
Radial artery			
Juxta-anastomotic diameter, mm	3.6 (2.9–4.2)	3.6 (3.0–4.0)	0.83
Flow, ml/min	456 (277–688)	406 (300–772)	0.81
Brachial artery			
Flow, ml/min	523 (342–985)	550 (417–1201)	0.79
3 mo			
Cephalic vein			
Juxta-anastomotic diameter, mm	4.2 (2.3–6.1)	4.9 (3.9–5.8)	0.43
Elbow diameter, mm	6.2 (4.7–6.9)	5.7 (4.4–6.3)	0.35
Mid upper arm diameter, mm	5.8 (2.8–4.5)	5.7 (3.6–6.2)	0.83
Radial artery			
Juxta-anastomotic diameter, mm	4.0 (2.1–5.0)	3.6 (3.0–4.6)	1.00
Flow, ml/min	546 (110–1037)	560 (334–970)	0.65
Brachial artery			
Flow, ml/min	800 (434–1485)	798 (479–1019)	0.60

IQR, interquartile range; LIPMAT, Liposomal Prednisolone to Improve Hemodialysis Fistula Maturation.

### Functional Outcomes

At the time of assessment of the functional outcomes, 54% of AVFs in the placebo arm and 69% in the liposomal prednisolone arm were successfully used for HD (*P* = 0.41). Seven patients (44%) in the liposomal prednisolone arm and 4 patients (31%) in the placebo group underwent an endovascular or surgical procedure to achieve RCAVF maturation. During follow-up, in the placebo and liposomal prednisolone groups, respectively 23% and 13% of RCAVFs had failed (*P* = 0.45). The functional outcome could not be determined for 6 patients, because of loss to follow-up (2 patients who moved abroad) or not initiating HD (Table 3).

**Table 3:** Effect of liposomal prednisolone on functional outcomes of RCAVF in 29 patients in the LIPMAT study

Functional outcome	Placebo (n = 13)	Liposomal prednisolone (n = 16)
AVF used		
Without procedures to improve maturation	3 (23)	4 (25)
With procedures to improve maturation	4 (31)	7 (44)
AVF not used		
Failed due to nonmaturation	3 (23)	2 (13)
Kidney transplantation	0 (0)	1 (6)
Did not reach ESRD	1 (8)	1 (6)
Deceased before ESRD	0 (0)	1 (6)
Loss to follow-up	2 (16)	0 (0)

Values are n (%).

AVF, arteriovenous fistula; ESRD, end-stage renal disease; LIPMAT, Liposomal Prednisolone to Improve Hemodialysis Fistula Maturation.

### Safety

No infusion reactions were observed except for 1 subject in the liposomal prednisolone arm who was known to have symptoms of orthostatic hypotension, and experienced mild dizziness without hypotension on postural change during the infusion. The incidence of symptoms related to progressive renal failure and cardiovascular events was similar in both treatment arms (Table 4).

**Table 4:** Adverse events reported in the LIPMAT study

Adverse events	Placebo (n = 13)	Liposomal prednisolone (n = 16)
AVF-related events		
Angiography/angioplasty	3	6
Revision surgery	1	0
Coiling or ligation of collateral veins	1	2
Hematoma or bleeding	2	1
New AVF within 3 mo	1	1
Nerve damage	1	0
Edema	1	0
Infusion-related events		
Orthostatic symptoms (no hypotension)	0	1
Renal and metabolic		
Fluid overload	3	2
Gout	1	0
Uremia (worsening)	1	0
Anemia (worsening)	1	1
Cardiovascular		
Atrial fibrillation/flutter	2	4
Myocardial infarction	1	2
Angina pectoris (worsening)	0	1
Intermittent claudication (worsening)	1	0
Infectious		
AVF site infection	0	1
Cellulitis (non-AVF site)	0	1
Upper airway infection including rhinosinusitis	0	2
Septicemia	0	1
Dental	1	0
Other		
Accidental injury	3	2
Fatigue and sleep disorders	4	4
Liver enzyme abnormalities	2	2
Hyperthyroidism	0	1
Hair loss	1	0
Intoxication	0	1
Aspecific thoracic pain	0	1
Constipation	0	1
Sunburn	0	1
Melanoma	1	0
Gastric pain	0	1
Hematoma non-AVF site	0	1
Urinary catheter placement	0	1

Myocardial infarction includes non-ST-elevation myocardial infarction.

AVF, arteriovenous fistula; LIPMAT, Liposomal Prednisolone to Improve Hemodialysis Fistula Maturation.

### Infections

In the liposomal prednisolone arm, 5 infections were observed in the 3 months of follow-up. One subject was treated with antibiotics due to erythema in the AVF arm, without fever or systemic symptoms. One subject experienced 2 episodes of mild rhinosinusitis that resolved without specific treatment. One subject died 72 days after AVF surgery, because of progressive fluid overload, complicated by septicemia from a possible catheter-related infection or pneumonia. In the placebo group, 1 subject experienced a dental abscess 3 months after AVF surgery.

## Discussion

In the LIPMAT study, we evaluated if liposomal prednisolone improves maturation of RCAVFs. The trial was terminated because of slow enrollment after inclusion of 30 of the 80 subjects initially aimed for. We present the study to investigate feasibility and to report preliminary outcomes. Liposomal prednisolone was safe and well-tolerated by patients with end-stage renal disease. No severe infusion reactions were observed and no severe infections were observed within the expected duration of effect of liposomal prednisolone. Liposomal prednisolone did not result in improved RCAVF maturation as measured by ultrasound at 6 weeks and 3 months after surgery. The 62% successful cannulation rate observed in the LIPMAT study was comparable to previous studies on functional AVF maturation.^1^^,^^7^ Although the nonsignificant result may be a mere result of a lack of power due to the small sample size, also no trend toward any difference between the treatment and control group was observed. Apart from a lack of statistical power, several factors might explain the lack of therapeutic efficacy of liposomal prednisolone to improve AVF maturation. First, the local concentration of liposomal prednisolone in the vessel wall of the AVF might not have been sufficient to exert a strong anti-inflammatory effect. The local accumulation of liposomal prednisolone could not be examined, as the AVFs could not be sacrificed for examination. In addition, no approved formulation of the compound was available to trace the liposomes in vivo in humans. Second, the inflammatory response in the RCAVF might have been too limited to induce significant local vascular accumulation of the liposomes. Previous clinical studies revealed substantial localization of liposomal prednisolone in the atherosclerotic arterial wall.^8^ As the prevalence of atherosclerosis was high in the LIPMAT subjects (Table 1), a significant proportion of liposomal prednisolone may therefore have accumulated in nontarget vessel walls instead of the AVF vein. In future studies, tissue samples of AVFs that failed early may be acquired during surgical revisions and analyzed for liposomal prednisolone content.

The extent and timing of venous inflammation after AVF surgery in humans is not fully known. To avoid potential detrimental effects on wound healing, liposomal prednisolone was not administered before surgery. As most of outward remodeling of AVFs has been shown to occur within the first 4 weeks after surgery,^9^ we aimed to cover this interval by administering the drug at day 1 and 15 after surgery. This might have been too short, with significant inflammation persisting at 4 weeks after surgery.

## Conclusion and Further Directions

The LIPMAT study was the first to study an anti-inflammatory strategy to improve AVF maturation in humans. We could not demonstrate a clinically significant impact on RCAVF maturation. Future studies are needed to elucidate the role of inflammation in AVF maturation and the clinical promise of liposomal formulations of anti-inflammatory drugs to promote AVF maturation.
