# Supplementary material for: A Randomized Trial of Liposomal Prednisolone (LIPMAT) to Enhance Radiocephalic Fistula Maturation: A Pilot Study
Source: Kidney Int Rep. 2020 Jun 5;5(8):1327–32. doi: 10.1016/j.ekir.2020.05.030 (PMC7403542; doi:10.1016/j.ekir.2020.05.030)
Supplement: Supplementary File (PDF) [file mmc1.pdf]

Supplementary materials

Inclusion and exclusion criteria

Inclusion criteria

In order to be eligible to participate in this study, a subject must meet all of the following criteria:

1. Patients who are scheduled for creation of a radiocephalic AVF for maintenance hemodialysis.
2. Male or female ≥ 18 years old.
3. Patients are able and willing to give written informed consent.

Exclusion criteria

A potential subject who meets any of the following criteria will be excluded from participation in this study:

1. Any concurrent illness, disability or clinically significant abnormality that may, as judged by the investigator, affect the interpretation of clinical efficacy or safety data or prevent the subject from safely completing the assessments required by the protocol.
2. Current participation in another interventional clinical trial or subjects who have received an investigational drug within 30 days prior to the baseline visit.
3. History of psychosis.
4. History of osteonecrosis.
5. Previous AVF in the ipsilateral arm.
6. Initial version: Current central venous catheter at the ipsilateral side.

After 2017-04-25: Current central venous catheter at the ipsilateral side with central venous obstruction.

7. Treatment with oral, rectal or injectable (including intra-articular) glucocorticoids (CS) within 6 weeks prior to baseline visit. Inhaled glucocorticoids are allowed. Topical steroids are allowed, however subjects should not have received more than 100 gram of a mild to moderate topical corticosteroid cream per week, 50 gram of a potent corticosteroid cream per week or 30 gram of a very potent topical corticosteroid cream per week in the 4 weeks prior to the baseline visit.
8. Treatment with immunosuppressant drugs. Treatment with NSAIDs.
9. Patients who are unlikely to adequately comply with the trial's procedures (due for instance to medical conditions likely to require an extended interruption or discontinuation, history of substance abuse or noncompliance).
10. Women who are lactating, pregnant (positive pregnancy test at baseline) or planning to become pregnant during the course of the study.
11. Unwillingness to use reliable and acceptable contraceptive methods throughout the study and till 3 months after last study medication except for female patients who are surgically sterile (bilateral tubal ligation, bilateral oophorectomy or hysterectomy) or at least 1 year postmenopausal.
12. Initial version: History of malignancy within 5 years before screening, with the exception of basal skin cell carcinoma which has been treated with no signs of recurrence.

    After 2016-05-02: Malignant disease, unless cured. Current prostate carcinoma without current or planned cytostatic therapy is allowed.
13. Uncontrolled diabetes mellitus.
14. Signs of active infection, requiring systemic treatment.

15. Positive Quantiferon test.

16. Subject with positive hepatitis panel (including hepatitis B surface antigen [HBsAg], and / or anti-hepatitis B core antibodies, and / or hepatitis C virus antibody [anti-HCV]).

17. History of anaphylaxis or severe allergic responses, including to radio-contrast agents.

18. Planned live-virus vaccinations.

19. Planned surgical interventions or planned elective hospital admissions within 6 weeks after AVF surgery. Planned hemodialysis sessions do not count as an exclusion criterion.

20. Abnormal hepatic function (ALT/AST or bilirubin > 2 x upper limit of normal) at the time of the screening visit.

21. Clinically significant out-of-range values on haematology panel, at discretion of the Principal Investigator.

22. Current substance abuse or alcohol abuse.

Methods

Study design

The Liposomal Prednisolone to Improve Hemodialysis Fistula Maturation (LIPMAT) was a phase 2, investigator-initiated, multi-center, double-blinded, randomized, placebo-controlled trial. Subjects were recruited in 11 participating hospitals in the Netherlands. Patients were eligible for enrolment if RCAVF creation was planned based on local hospital protocols, including a baseline ultrasound examination, as recommended by the Dutch Vascular Access Guidelines (S1). Treating physicians identified eligible patients based on the in- and exclusion criteria (Supplementary Materials). Patients who provided written informed consent were then assessed for eligibility at a screening visit by the investigators, using medical history, physical examination and laboratory investigations. If an RCAVF could be successfully created, patients were enrolled and randomized stratified per hospital 1:1 to two infusions of each 150mg liposomal prednisolone or matching placebo in 500ml normal saline. Treatments were administered at 1 (±1) day and 15 (±2) days after surgery. Although the plasma half-life of liposomal prednisolone is 3 days, previous studies in humans revealed a therapeutic effect of 2 weeks after a single dose of 150 mg liposomal prednisolone (S2). Therefore, we anticipated that the treatment regime in our study would results in an anti-anti-inflammatory effect that would last 4 weeks. All patients were pre-treated with paracetamol and clemastine before each infusion to mitigate any allergic responses. Blinding methods have been described previously in detail, the investigators and patients were blinded to treatment allocation (6). The protocol was approved by the ethics committee of the Leiden University Medical Center and the Institutional Review Boards of all participating hospitals and the study was performed in accordance with the principles of the Declaration of Helsinki.

End points

The primary end point was the juxta-anastomotic diameter of the cephalic vein, measured by ultrasonography at 1cm downstream from the arteriovenous anastomosis at 6 weeks (± 5 days) after surgery. Secondary end points were the diameter of the cephalic vein at the elbow and mid upper arm and blood flow in the upstream radial and brachial arteries at 6 weeks and 3 months (± 14 days). The 6-week and 3-month time points chosen for AVF evaluation are similar to other studies evaluating the effect of pharmaceutical interventions on AVF maturation (S3, S4). Ultrasound examinations were performed by qualified personnel in the participating hospitals. In case of AVF occlusion, diameters and flow were analysed as 0 mm and 0 ml/min respectively. Adverse events were recorded up to 3 months after surgery. Adverse events were classified as 'severe' if these met the criteria for Serious Adverse Events according to Good Clinical Practice guidelines, stating that an adverse event is serious if it is fatal, and/or is life-threatening for the subject, and/or makes hospital admission or an extension of the admission necessary, and/or causes persistent or significant invalidity or work disability, and/or manifests itself in a congenital abnormality or malformation, and/or could, according to the person that carries out the research, have developed to a serious undesired medical event, but was however prevented due to premature interference. Functional outcomes were assessed in December 2018 for all subjects.

Statistical analysis

In a pilot cohort, a 1.5 mm standard deviation of the 6-week distal cephalic vein diameter was observed. A difference of 1.0 mm between the treatment and control group was considered clinically relevant. The sample size was calculated at 40 patients per group, allowing for a drop-out of 10%. The non-normally distributed end points were described as median and interquartile range (IQR) and were tested for significance using the Mann-Whitney U-test. The

proportions of AVF occlusions were reported as percentages per treatment group. The study was not powered to demonstrate differences in AVF occlusions, side effects and functional outcomes and no statistical analysis was performed for these parameters.

Supplementary references

S1. Guideline vascular access (“Richtlijn vaattoegang”) - Dutch Federation of Nephrology (“Nederlandse Federatie voor Neffrologie”). 2009.

S2. van Assche G, Rutgeerts P, Ferrante M, Noman M, Fidder H, Oldenburg B, et al. Safety and efficacy of a novel IV targeted pegylated liposomal prednisolone product (Nanocort): results from a phase 2a study in patients with active ulcerative colitis. In: European Crohn’s and Colitis Organisation. 2016.

S3. Peden EK, Leeser DB, Dixon BS, El-Khatib MT, Roy-Chaudhury P, Lawson JH, et al. A Multi-center, Dose-escalation Study of Human type I Pancreatic Elastase (PRT-201) Administered after Arteriovenous Fistula Creation. J Vasc Access. 2013 Apr 20;14(2):143–51.

S4. Dember LM, Beck GJ, Allon M, Delmez JA, Dixon BS, Greenberg A, et al. Effect of clopidogrel on early failure of arteriovenous fistulas for hemodialysis: a randomized controlled trial. JAMA. 2008 May 14;299(18):2164–71.
